# The Combination of Functional Magnetic Stimulation and Low-Frequency Therapeutic Ultrasound for Body Shaping: Preliminary Case Reports

**DOI:** 10.7759/cureus.78121

**Published:** 2025-01-28

**Authors:** Ana Kristina Klancic, Marko Klancic

**Affiliations:** 1 Therapeutic Department, KlanMedic Diagnostic and Therapeutic Center, Nova Gorica, SVN

**Keywords:** body shaping, cavitation, functional magnetic stimulation, gluteus, ultrasound

## Abstract

Functional magnetic stimulation (FMS) is commonly used to accelerate the healing process and alleviate pain. Recently, it has been shown to be useful for non-invasive body-shaping techniques. Low-frequency therapeutic ultrasound has been proven to be a safe, non-invasive alternative to liposuction. This report discusses a few cases to compare the effects of FMS with the combination of FMS with low-frequency therapeutic ultrasound on muscle thickness, volume, and subcutaneous adipose tissue thickness which can increase muscle strength and loss of unwanted fat.

Four healthy Caucasian female volunteers aged between 42 and 45 years received 10 sessions of FMS using the Tesla Former prestige device (Iskra Medical, Otoče, Slovenia). Additionally, three volunteers (out of four) received four sessions of low-frequency therapeutic ultrasound with the Sonic Shaper device (Iskra Medical). The efficacy of the treatments was evaluated through ultrasound imaging and tensiomyography (TMG). Patients 1 and 2 received the application of FMS on the gluteal area and Sonic Shaper on the gluteal adipose tissue. However, patient 4 received the combination treatment on the rectus abdominalis, and patient 3 received only FMS treatment on the gluteus maximus (GMx) and gluteus medius (GMe) instead.

The measurements collected after the last session showed significant improvements in each patient. Post-treatment measurement of left/right gluteus maximus diameter for patients 1, 2, and 3 showed an increase compared to the baseline. Patient 4, who received combination treatment on the rectus abdominis muscle (RAb), also showed an increase in rectus abdominis diameter. The study shows that FMS increases muscle volume, whereas low-frequency therapeutic ultrasound reduces localized subcutaneous adipose tissue thickness. Combining these two non-invasive treatments may be a promising, safe, and effective intervention for body shaping and countering.

## Introduction

Electric stimulation of muscles and nerves has existed since 1896 when d’Arsonval used a strong, time-varying current to stimulate living tissue. His experiments showed the potential of nerve stimulation to contract muscles, and research on the subject was carried out throughout the 20th century. In 1965, the first successful muscle twitching was obtained, and, 10 years later, transcranial magnetic stimulation was applied for clinical purposes [1]. The last two decades have witnessed an upswing in the popularity of gluteal reconstructive surgery, with personalized exercises for strengthening the gluteal muscles and specific exercises all prescribed for the treatment of the lower back as well as lower extremities. This increase in demand and interest has influenced aesthetic norms and physical exercise and is attributed to refined and improved contouring techniques [2]. As a fast-growing field, many new surgical procedures have been adapted to achieve satisfactory and refined outcomes. However, increased engagement in physical activities, such as leisure time and professional sports, can lead to sport-related injuries [3]. Therefore, many prefer the use of augmentation techniques as a safer alternative [4-6].

Muscle size increases when a person continually challenges the muscles to deal with higher levels of resistance or weight. This process is known as muscle hypertrophy. Muscle hypertrophy occurs when the fibers of the muscles sustain microdamage or injury. The body repairs damaged fibers by fusing them, which increases the mass and size of the muscles. Certain hormones, including testosterone, human growth hormone, and insulin growth factor, also play a role in muscle growth and repair [7]. They may also be used and frequently prescribed for the treatment of lower back and lower extremity pathology [4, 8].

Traditionally, functional magnetic stimulation (FMS) has been used in gynecology and physiotherapy to accelerate the healing process and alleviate pain by strengthening the pelvic floor muscles [9-11]. Recently, FMS has been used as a non-invasive body-shaping technique [12-14]. The preceding technique, i.e., electromyostimulation, is sometimes used for voluntary exercise in athletes to improve their fitness. However, it does not penetrate deep enough, rarely produces satisfactory results, and provides the best results only if the whole body is subjected to treatment [15]. Most literature documenting FMS as a body-shaping technique uses cooperative standardized images, patient satisfaction questionnaires, or circumference measurements as their method for monitoring the results.

To our knowledge, no previous study has investigated the cumulative effects of FMS and low-frequency therapeutic ultrasound on outcomes. It is known that low-frequency therapeutic ultrasound delivers an energy signature through the skin for adipose tissue disruption. Adipose tissue disruption releases triglycerides into the extracellular spaces and bloodstream. The released triglycerides are now readily available for metabolic processes. This has been proven to be a safe, non-invasive alternative to liposuction and an efficient way to reduce the circumference of the treated area [16]. We hypothesized that combining FMS and low-frequency therapeutic ultrasound will improve body shaping by strengthening the skeletal muscles and removing localized adipose tissue build-up [10,15,16]. 

This case report aimed to explore the effects of FMS and the combination of FMS and low-frequency therapeutic ultrasound on muscle thickness and volume, as well as subcutaneous adipose tissue thickness. We also aimed to investigate the safety and efficiency of both treatment methods in body shaping.

## Case presentation

Four healthy female volunteers took part in the case study, with an age range from 42 to 45. Three patients received FMS and ultrasound treatments. Of these three patients, two received treatment on the gluteus and one on the abdomen. One patient received only FMS on the gluteus muscle. A written, informed consent was obtained from the participants before the study. The study was conducted according to the Declaration of Helsinki.

Gluteus and hamstring muscle magnetic stimulation

All patients received FMS 45 minutes a day, every second day (three times a week) for 10 sessions, using the Tesla Former prestige device (Iskra Medical, Otoče, Slovenia). During the treatment, two XXL applicators were placed on the gluteal area and two XL applicators were placed on the hamstring area. The program Muscle Strengthening II was used, which concurrently activated the gluteus applicators at the frequency of 30 Hz, followed by the hamstring applicators simultaneously at 30 Hz. One patient received FMS on the abdomen. An XL applicator was placed on the abdomen for this patient and two XL applicators were placed on the thighs. The program Abdomen Thighs I was used. The applicators were connected to the body using fixing straps. The intensity was set to fit the individual tolerability level of each participant. All individuals were able to painlessly handle 100% of the total device magnetic field intensity of 3 T at every session for the entire duration. Potential adverse events during or after the treatment were monitored in both participants.

Low-frequency therapeutic ultrasound of the gluteus and hamstring area

In addition to the 10 FMS sessions, three participants received low-frequency therapeutic ultrasound treatment. Patients 1 and 2 were treated for 40 minutes on the gluteus maximus and 20 minutes on each thigh, once a week spanning four sessions, using the Sonic Shaper device (Iskra Medical, Otoče, Slovenia). Patient 3 was treated with Sonic Shaper for 40 minutes on the abdomen, 20 minutes on each side, once a week for four sessions. Both the FMS and the Sonic Shaper treatments started on the same day. The treatment was carried out using a large Sonic Combo applicator. The parameters were set to 100% of low-frequency ultrasound intensity, and the vacuum pulsed between 60 and 120 hPa. Potential adverse events during or after the treatment were monitored.

Evaluation methodology

All participants were subjected to diagnostic ultrasound impinging of the treated areas. The diagnostic ultrasound was carried out at KLANMEDIC d.o.o., - Diagnostični in Terapevtski Center, Slovenia. The muscle diameter and adipose tissue thickness were measured using the diagnostic ultrasound, with MyLab9 exp Esaote linear probe (Esaote SpA, Genoa, Italy), from L4-L15, set to operate at 4-15 MHz. The ultrasound measurement was repeated twice before the start of treatment and once after all the treatments were completed.

The participant treated only with FMS was also subjected to tensiomyography (TMG) using a TMG device (TMG d.o.o, Ljubljana, Slovenia) to observe the difference in maximal displacement of the probe. The increase in deviation amplitude points to increased muscle volume and the ability to activate a more significant number of motor units during contracting.

Findings

**Figure 1:**
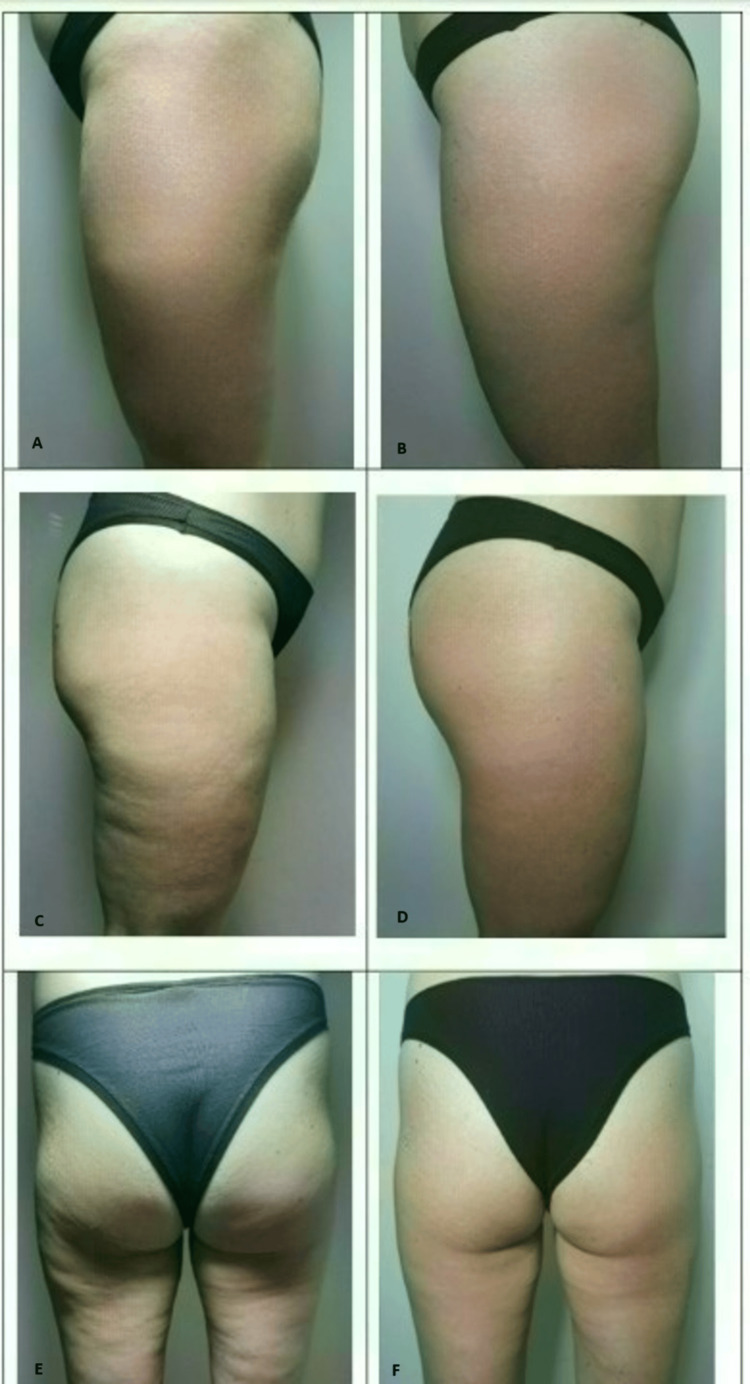
Comparison of images of the patient treated with both FMS and Sonic Shaper at the baseline and at the end of the protocol Baseline (A, C, and E). At the end of the protocol (B, D, and F) FMS: functional magnetic stimulation

The measurements taken with a diagnostic ultrasound are summarized in Table 1. Figure 1 shows the before and after images following the combined treatment. 

**Table 1:** Results of diagnostic ultrasound measurements of the gluteus area and abdomen Patients 1, 3, and 4 were treated with the combination of FMS and Sonic Shaper, and the patient two was treated with FMS only. Patients one, two and three were treated on the gluteal area. Patient four was treated on the abdomen FMS: functional magnetic stimulation; L: left; R: right

		Baseline diameter (mm)	End diameter (mm)	Difference betwwen end/baseline (%)
Gluteus maximus - L	Patient no. 1	42	54	28
	Patient no. 2	25	42	68
	Patient no. 3	38	48	26
Gluteus maximus - R	Patient no. 1	33	48	45
	Patient no. 2	22	38	53
	Patient no. 3	33	41	24
Gluteus medius - L	Patient no. 1	22	25	14
	Patient no. 2	18	22	22
Gluteus medius - R	Patient no. 1	20	27	35
	Patient no. 2	20	26	30
Rectus abdominalis - L	Patient no. 4	11	14	19
Rectus abdominalis - R	Patient no. 4	10	13	25
Adipose tissue - L	Patient no. 1	23	17	- 26
	Patient no. 3	25	17	-30
	Patient no. 4	18	13	-24
Adipose tissue - R	Patient no. 1	28	27	- 4
	Patient no. 3	18	14	-20
	Patient no. 4	14	12	-10

Patient 1 received a multimodal treatment of FMS and Sonic Shaper. The devices were applied on the gluteus maximus (GMx) and gluteus medius (GMe) muscles. Measurements before treatment of the muscle diameter were 42 mm for the left GMx, 33 mm for the right GMx, 22 mm for the left GMe, and 20 mm for the right GMe. After the last session, new measurements were taken. The left Gmx was measured at 54 mm, which indicated a 12 mm (or 28%) increase, right GMx was at 48 mm, a 15 mm (or 45%) increase, left GMe was at 25 mm, a 3mm (or 14%) increase, and finally the right GMe was measured at 27 mm, a 7 mm (or 35%) increase in diameter.

Patient 2 received a multimodal treatment of FMS and the Sonic Shaper on her gluteal area, more accurately on the GMx. Starting measurements of the muscle diameter were 25 mm on the left GMx and 22 mm on the right GMx. The diameter of adipose tissue on the left buttock was 25 mm, while it was 18 mm on the right. The final result was 42 mm (17 mm or 68% increase) on the left GMx, 34 mm (12 mm or 53% increase) on the right GMx for the muscle diameter, and 17 mm (8 mm or -30% decrease) for the left and 14 mm (4mm or -20% decrease) for the right side of adipose tissue.

Patient 3 received only FMS applications on both sides of GMx and GMe. Starting measurements that were taken before treatment were 38 mm for the left GMx, 33 mm for the right GMx, 18 mm for the left GMe, and 20 mm for the right GMe. After the last treatment, new measurements were taken, which were as follows: 48 mm (10 mm or 26% increase) on the left GMx, 41 mm (8 mm or 24% increase) in the right GMx, 22 mm (4 mm or 22% increase) for the left GMe and 26 mm (6 mm or 30% increase) for the right GMe.

Patient 4, on the other hand, received a multimodal treatment of FMS and Sonic Shaper on the abdominal area, more accurately on the rectus abdominis muscle (RAb). Baseline measurements were as follows: 11 mm for the left side and 10 mm for the right side of the muscle; adipose tissue was measured at 18 mm for the left and 14 mm for the right side. The results were as follows: 14 mm (3 mm or 19% increase) on the left RAb and 13 mm (3 mm or 25% increase) on the right RAb regarding muscle diameter and 13 mm (5 mm or 24% decrease) on the left side RAb and finally 12 mm (2 mm or 10% decrease) for the right RAb regarding the adipose tissue.

**Figure 2:**
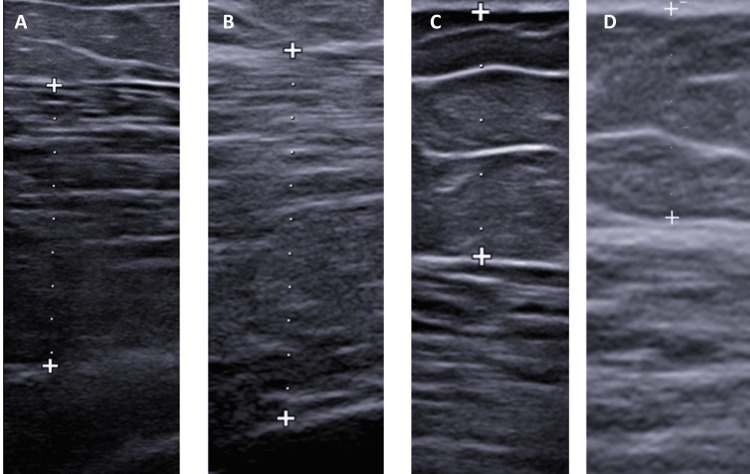
Diagnostic ultrasound images at the baseline and at the end of the protocol Diagnostic ultrasound images of the left gluteus maximus of the patient treated with both FMS and Sonic Shaper at the baseline (A) and at the end of the protocol (B); the plus signs and the white dots mark the diameter of the gluteus maximus. Diagnostic ultrasound images of the subcutaneous adipose tissue over the left gluteus maximus of the patient treated with both FMS and Sonic Shaper at the baseline (C) and at the end of the protocol (D); the plus signs and the white dots mark the thickness of the subcutaneous adipose tissue FMS: functional magnetic stimulation

Figure 2 presents the ultrasound images at the baseline and at the end of the protocol.

## Discussion

The findings of this case series highlight the potential efficacy of combining FMS and low-frequency therapeutic ultrasound (Sonic Shaper) for body shaping, with the results indicating significant improvements in muscle diameter and reductions in localized adipose tissue. These preliminary outcomes align with previous studies, such as those by Jacob et al. (2020) [17], which reported favorable outcomes following high-intensity focused electromagnetic stimulation for postpartum abdominal wall toning. The absence of significant side effects in our cases further endorses the safety profile of this approach.

The increase in muscle diameter observed across all patients, ranging from 14% to 68%, suggests that FMS facilitates muscle hypertrophy. Notably, patients who underwent the combined treatment of FMS and Sonic Shaper showed superior muscle growth compared to those receiving FMS alone. For instance, patient 1 achieved a 45% increase in the right GMx, while patient 2 achieved a 68% increase in the left GMx, demonstrating the added benefit of low-frequency ultrasound in enhancing the outcomes of FMS. This enhancement may be attributed to the synergistic effect of the Sonic Shaper, which improves local circulation and optimizes tissue conditions for muscle development.

Localized adipose tissue reduction in patients treated with both FMS and Sonic Shaper further underscores the potential of this multimodal approach. For example, patient 2 experienced a 30% reduction in left gluteal adipose tissue thickness, while patient 4 exhibited a 24% reduction in left abdominal adipose tissue thickness. These results are in line with the known effects of low-frequency ultrasound, which enhances lipolysis by delivering mechanical vibrations that disrupt adipocyte membranes without damaging surrounding tissues. The absence of necrosis or apoptosis supports the safety of this technique, offering a compelling alternative to more invasive procedures like liposuction. Interestingly, patient 3, who underwent FMS alone, still showed substantial muscle growth (e.g., a 26% increase in left GMx). This indicates that while FMS alone is effective in promoting muscle hypertrophy, the addition of low-frequency ultrasound significantly amplifies the outcomes, particularly for combined muscle toning and adipose tissue reduction.

Our results also align with findings from TMG, which demonstrated increased maximal displacement in muscles treated with FMS, further confirming muscle hypertrophy and improved neuromuscular function. These results suggest that FMS-induced muscle contractions mimic the effects of high-intensity resistance training, leading to enhanced muscle volume and function. Previous studies have also supported these findings [18,19].

While the outcomes of this case series are promising, the small sample size and lack of a control group limit the generalizability of the findings. A larger-scale, double-blind randomized controlled trial is required to validate the efficacy and safety of this combined approach. Such a trial should ideally include three groups: a placebo group (sham FMS and sham ultrasound), an FMS-only group (real FMS and sham ultrasound), and a combined treatment group (real FMS and real ultrasound). This design would allow for a more robust comparison of the relative contributions of each modality.

## Conclusions

This report provides preliminary evidence that the combination of FMS and low-frequency therapeutic ultrasound is a safe and effective non-invasive method for body shaping. FMS appears to promote muscle hypertrophy, while low-frequency ultrasound effectively reduces localized adipose tissue. Together, these modalities offer a synergistic approach that could serve as an alternative to traditional surgical interventions. Future research with larger sample sizes and rigorous study designs is warranted to confirm these findings and further explore the mechanisms underlying this promising multimodal treatment.

## References

[1] Geddes LA (1991). History of magnetic stimulation of the nervous system. J Clin Neurophysiol.

[2] Centeno RF (2006). Gluteal aesthetic unit classification: a tool to improve outcomes in body contouring. Aesthet Surg J.

[3] Huebner M, Ma W (2022). Health challenges and acute sports injuries restrict weightlifting training of older athletes. BMJ Open Sport Exerc Med.

[4] Reiman MP, Bolgla LA, Loudon JK (2012). A literature review of studies evaluating gluteus maximus and gluteus medius activation during rehabilitation exercises. Physiother Theory Pract.

[5] Tojima S, Anetai H, Koike K, Anetai S, Tokita K, Leigh C, Kumaratilake J (2022). Gross anatomy of the gluteal and posterior thigh muscles in koalas based on their innervations. PLoS One.

[6] Leite MJ, Pinho AR, Silva MR, Lixa JC, Madeira MD, Pereira PG (2022). Deep gluteal space anatomy and its relationship with deep gluteal pain syndromes. Hip Int.

[7] Loenneke JP, Rossow LM, Fahs CA, Thiebaud RS, Grant Mouser J, Bemben MG (2017). Time-course of muscle growth, and its relationship with muscle strength in both young and older women. Geriatr Gerontol Int.

[8] Oranges CM, Tremp M, di Summa PG, Haug M, Kalbermatten DF, Harder Y, Schaefer DJ (2017). Gluteal augmentation techniques: a comprehensive literature review. Aesthet Surg J.

[9] Fujishiro T, Takahashi S, Enomoto H, Ugawa Y, Ueno S, Kitamura T (2002). Magnetic stimulation of the sacral roots for the treatment of urinary frequency and urge incontinence: an investigational study and placebo controlled trial. J Urol.

[10] Ullah I, Arsh A, Zahir A, Jan S (2020). Motor relearning program along with electrical stimulation for improving upper limb function in stroke patients: a quasi experimental study. Pak J Med Sci.

[11] Zeb A, Arsh A, Bahadur S, Ilyas SM (2018). Effectiveness of transcutaneous electrical nerve stimulation in management of neuropathic pain in patients with post traumatic incomplete spinal cord injuries. Pak J Med Sci.

[12] Jacob CI, Paskova K (2018). Safety and efficacy of a novel high-intensity focused electromagnetic technology device for noninvasive abdominal body shaping. J Cosmet Dermatol.

[13] Leone A, Piccolo D, Conforti C, Pieri L, Fusco I (2021). Evaluation of safety and efficacy of a new device for muscle toning and body shaping. J Cosmet Dermatol.

[14] Fabi S, Dover JS, Tanzi E, Bowes LE, Tsai Fu F, Odusan A (2021). A 12-week, prospective, non-comparative, non-randomized study of magnetic muscle stimulation for improvement of body satisfaction with the abdomen and buttocks. Lasers Surg Med.

[15] Stöllberger C, Finsterer J (2019). Side effects of and contraindications for whole-body electro-myo-stimulation: a viewpoint. BMJ Open Sport Exerc Med.

[16] Mahmoud ELdesoky MT, Mohamed Abutaleb EE, Mohamed Mousa GS (2016). Ultrasound cavitation versus cryolipolysis for non-invasive body contouring. Australas J Dermatol.

[17] Jacob CI, Rank B (2020). Abdominal remodeling in postpartum women by using a high-intensity focused electromagnetic (HIFEM) procedure: an investigational magnetic resonance imaging (MRI) pilot study. J Clin Aesthet Dermatol.

[18] Mofid MM, Teitelbaum S, Suissa D, Ramirez-Montañana A, Astarita DC, Mendieta C, Singer R (2017). Report on mortality from gluteal fat grafting: recommendations from the ASERF Task Force. Aesthet Surg J.

[19] Nisticò SP, Bonan P, Coli F (2022). A new protocol to treat abdominal subcutaneous fat combining microwaves and flat magnetic stimulation. Bioengineering (Basel).

